# A novel internal fixation system for sacral fractures: a finite element biomechanical study

**DOI:** 10.3389/fbioe.2026.1871603

**Published:** 2026-07-10

**Authors:** Bin Chen, Xinbo Gu, Beiyang Wang, Ningning Shen, Mingming Kang, Bo Zhang, Haiyu Sun, Huan Wang, Yonghong Zhang

**Affiliations:** 1 Second Clinical Medical College, Shanxi Medical University, Taiyuan, China; 2 Department of Orthopaedics, Second Hospital of Shanxi Medical University, Taiyuan, China; 3 Third Hospital of Shanxi Medical University, Shanxi Bethune Hospital, Shanxi Academy of Medical Sciences, Tongji Shanxi Hospital, Taiyuan, China

**Keywords:** biomechanics, finite element analysis, internal fixation, novel fixation system, posterior pelvic ring, sacral fractures

## Abstract

**Background:**

Sacroiliac joint screw (SIJS) fixation is widely regarded as the gold standard for treating unstable posterior pelvic ring injuries. However, the technique presents notable challenges, including a high risk of neurovascular injury and a steep learning curve. This study aimed to introduce a novel Posterior Internal Fixation (P-INFIX) system and evaluate its biomechanical performance in the treatment of Denis zone I sacral fractures using finite element analysis.

**Methods:**

A three-dimensional pelvic model—cortical bone, cancellous bone, and cartilage—was reconstructed from computed tomography data of a healthy volunteer. A Denis zone I sacral fracture was simulated and stabilized using either the P-INFIX system (bilateral pedicle screws connected by a transverse Kirschner wire) or the standard SIJS. Finite element analysis was conducted under simulated sitting, supine, and lateral decubitus conditions to assess fracture fragment displacement and implant stress distribution.

**Results:**

The P-INFIX system provided fracture stability comparable to SIJS under most testing conditions. Although SIJS demonstrated a slight statistical advantage in limiting lateral fragment displacement (For example, Z2 in the sitting position: 0.13 mm vs. 0.18 mm, *P* = 0.001), all measured displacements were far below the clinically significant threshold (<1.3 mm). In terms of implant performance, the peak stress in P-INFIX (sitting: 141 MPa; supine: 320 MPa) was higher than that in SIJS; however, it remained well within the safe range below the yield strength of titanium alloy (1,050 MPa).

**Conclusion:**

The novel P-INFIX system demonstrated biomechanical stability comparable to the gold standard SIJS in managing Denis zone I sacral fractures. Given its potential to reduce surgical risks and simplify the procedure through a “safe channel” lateral to the posterior superior iliac spine, P-INFIX represents a promising and more accessible alternative fixation technique.

## 1 Introduction

The posterior pelvic ring is the central load-bearing structure of the human body, providing up to 70% of the mechanical stability of the pelvis (Bi et al., 2016). As its main component, the sacrum is involved in 17%–45% of pelvic fractures (Ma et al., 2023). These fractures are typically caused by high-energy trauma and frequently present as unstable injuries (Krassnig et al., 2021). Failure to achieve timely and effective stabilization can result in serious complications such as intractable pain, lower limb length discrepancy, and long-term functional disability (Lee et al., 2023). Currently, the primary fixation techniques for unstable posterior pelvic ring injuries (Zhao et al., 2024)include sacroiliac joint screws (SIJS), tension band plates, and transiliac–lumbar fixation.

SIJS fixation is considered the gold standard for managing these injuries (Hartensuer et al., 2021). Although this technique offers advantages—such as minimal invasiveness, limited soft-tissue dissection, and a low postoperative infection rate—it also has notable limitations. First, there is a considerable risk of neurovascular injury, with reported incidence rates ranging from 3% to 16% (Hoernschemeyer et al., 2017). For example, injury to the superior gluteal artery can lead to life-threatening hemorrhage. Second, SIJS requires advanced surgical expertise and a steep learning curve (Yuan et al., 2025), and repeated intraoperative fluoroscopy to confirm screw trajectory. This increases radiation exposure to the patient and surgical team, and also raises the risk of iatrogenic injury (Hinsche et al., 2002). Although navigation and robotic assistance (Liu et al., 2023) can improve placement accuracy, their high cost limits widespread use in most primary hospitals. Furthermore, early postoperative weight-bearing or functional exercise may cause screw loosening, migration, or even fracture, resulting in implant failure (Kim et al., 2016). Other surgical techniques have their own drawbacks. Posterior sacral locking plates, for example, are relatively easy to implant but are associated with a high incidence of wound infection and necrosis due to the thin soft-tissue coverage over the posterior sacrum (Asavamongkolkul and Waikakul, 2012). Repeated intraoperative contouring of the plate to match the bony anatomy can deform locking holes and weaken the construct’s mechanical strength. Conversely, lumbopelvic fixation enhances stability; however, it partially sacrifices lumbar mobility as a tradeoff (Cho et al., 2023).

To overcome the limitations of these existing fixation techniques, our team developed a novel minimally invasive posterior internal fixation (P-INFIX) system based on clinical experience for treating sacral fractures and sacroiliac joint dislocations. Inspired by the anterior pelvic INFIX technique (Vigdorchik et al., 2012), this design innovatively adapts it for posterior application using a “safe channel” (Smucker et al., 2010) located lateral to the posterior superior iliac spine (PSIS) for screw insertion. This approach simplifies the procedure, reduces the learning curve, minimizes reliance on continuous intraoperative fluoroscopy, and potentially lowers the risk of neurovascular injury.

To evaluate the biomechanical performance of this new fixation system, This study first established and validated the effectiveness of the pelvic model (Fu et al., 2014), and subsequently constructed a Denis Type I sacral fracture model on this basis. The mechanical behavior of the P-INFIX system was compared with that of the gold standard SIJS under simulated physiological loading conditions—specifically, sitting, supine, and lateral decubitus positions. The primary evaluation parameters included fracture fragment stability and stress distribution within the implants, providing essential biomechanical evidence to support its clinical translation.

## 2 Materials and methods

### 2.1 Surgical procedure of the novel P-INFIX system

**FIGURE 1:**
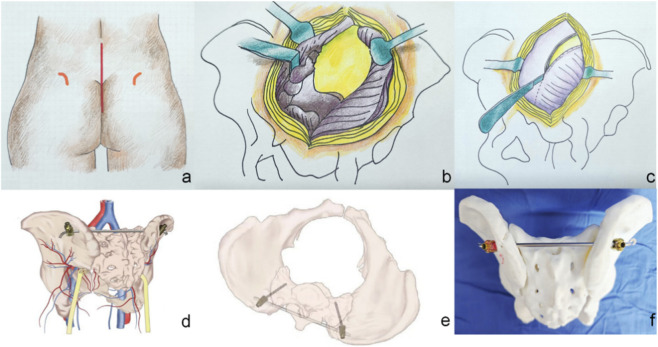
Surgical approach and model diagram of P-INFIX. **(a–c)** Surgical position and approach of P-INFIX. **(d)** Lateral view of P-INFIX. **(e)** Top view of P-INFIX. **(f)** 3D-printed model diagram of P-INFIX.

In the future, assuming this technique is applied clinically, the surgical procedure can be performed as follows: after induction of anesthesia, the patient was placed in the prone position. A curved incision approximately 5 cm in length was made 1–2 cm lateral to the bilateral PSIS. The skin and subcutaneous tissues were sequentially incised to expose the gluteal fascia. Blunt dissection was performed along the PSIS and the lateral border of the iliac wing to retract the gluteus maximus and medius muscles laterally, fully exposing the outer cortex of the ilium. Within the exposed area on each side, a pedicle screw (6.5 mm in diameter, 50 mm in length) was inserted with a trajectory oriented at 30° lateral divergence and 20° caudal inclination relative to the horizontal plane. After anatomical reduction of the sacral fracture was achieved using pelvic reduction forceps, a 4.0 mm transverse Kirschner wire (K-wire) was introduced through the predrilled channel in the tail of one screw. The wire was advanced medially along the iliac cortex, closely adhering to the bone surface until it reached the contralateral iliac cortex. The ends of the K-wire were trimmed to the appropriate length, bent, and securely seated in the tails of the bilateral screws. Finally, the tail caps were tightened to lock the entire internal fixation system in place (Figure 1).

### 2.2 Establishment of the pelvic finite element model

**FIGURE 2:**
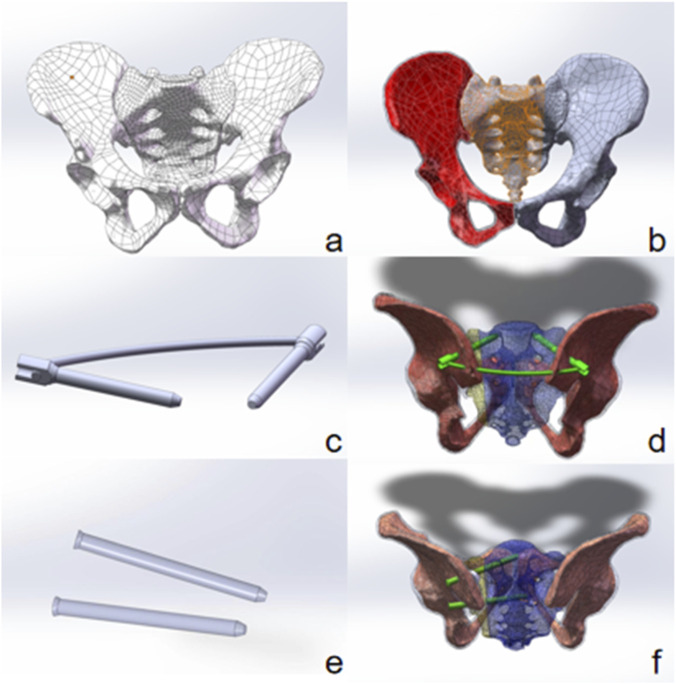
Establishment of finite element models of pelvis and internal fixation. **(a)** Three-dimensional model of normal pelvis. **(b)** Three-dimensional model of Denis type I sacral fracture. **(c,d)** P-INFIX device and fixation model. **(e,f)** SIJS device and fixation model.

This study was approved by the Ethics Committee of the Second Hospital of Shanxi Medical University. Informed consent was obtained from all participants, and all experiments were performed in accordance with the relevant guidelines and regulations. The model was constructed using pelvic computed tomography (CT) data (GE Revolution CT, United States; slice thickness, 0.625 mm) from a healthy adult male volunteer (28 years old, 170 cm, 70 kg). The scanning range extended from 4 cm above the superior border of the first sacral vertebra to the bilateral greater trochanters, with the lower limbs maintained in a neutral position during scanning. The volunteer had no history of pelvic or acetabular trauma, deformity, tumor, infections, or osteoporosis. DICOM-formatted CT data were imported into Mimics 21.0 (Materialise, Belgium). Bone structures were first extracted using threshold segmentation (226–3051 HU). Manual editing and region growing were then performed to isolate the sacrum and hip bones while removing the lumbar vertebrae and femurs. After denoising and repairing the preliminary model, it was exported in STL format and processed in Geomagic Wrap 2017 (3D Systems, United States) for surface optimization. The STL file was subsequently processed in Geomagic Wrap 2017 (3D Systems, United States) via the removal of spikes and holes, mesh repair, followed by contour line delineation and surface patch construction. The cortical bone model was obtained through offset and Boolean operations, with a thickness of 2 mm (Vigdorchik et al., 2012). Subsequently, the internal cancellous bone solid was constructed and finally exported in STP format. Bone assembly was completed using SolidWorks 2022 (Dassault Systèmes, France). Cartilage models were created for the bilateral sacroiliac joint surfaces and the pubic symphysis based on the CT images. After confirming the absence of interference, the final three-dimensional pelvic geometric model—comprising cortical bone, cancellous bone, and cartilage—was obtained (Figure 2a).

### 2.3 Construction and effectiveness validation of the ligament in finite element model

The three-dimensional pelvic model was imported into the finite element analysis software ANSYS 2024R1 (ANSYS, Inc., United States). A elastic material model was used to assign material properties to the cortical bone, cancellous bone, and cartilage based on parameters reported in previous studies (Li D. et al., 2023). Table 1 Major pelvic ligaments—including the anterior sacroiliac, posterior sacroiliac, sacrotuberous, sacrospinous, superior pubic, and arcuate pubic ligaments—were simulated using spring elements, with stiffness values defined according to established literature (Shi et al., 2014). (Table 2; Supplementary Figure S1) The model was discretized using tetrahedral elements with a global element size of 3 mm. The final pelvic finite element model consisted of 270,092 nodes and 160,171 elements. Model validation was conducted through two complementary approaches:

- Geometric validation:

**TABLE 1:** Material properties of FEA models.

Material	Elastic modulus (MPa)	Poisson’s ratio
Cortical bone	17,000	0.3
Cancellous bone	150	0.2
Pubic symphysis cartilage	5	0.495
Sacroiliac joint cartilage	54	0.4
Pedicle screw	12,000	0.3
K-wire	20,000	0.2
Iliac screw	12,000	0.4

**TABLE 2:** Properties of pelvic ligament models.

Ligaments	Stiffness (N/mm))	Quantity
Superior pubic ligament	500	1
Arcuate pubic ligament	500	1
Anterior sacroiliac ligament	700	8
Posterior sacroiliac ligament	1,400	4
Sacrotuberous ligament	1,500	8
Sacrospinous ligament	1,400	8

Following the methodology described in reference (Song et al., 2022), ten key anatomical landmarks were identified on the model: (a) distance between the bilateral highest points of the sacroiliac joints, (b) anteroposterior diameter of the sacral base, (c) distance from the sacral promontory to the coccyx tip, (d) distance from the iliac crest to the greater sciatic notch, (e) distance between the anterior superior iliac spine (ASIS) and the PSIS on the same side, (f) transverse diameter of the acetabular fossa, (g) longitudinal diameter of the acetabular fossa, (h) distance from the ischial tuberosity to the midpoint of the pubic symphysis, (i) distance from the iliac crest to the ischial tuberosity, and (j) distance between the ASIS and anterior inferior iliac spine on the same side. The measured distances between these landmarks were compared with those obtained from the original CT data, and only minimal deviation was observed, confirming the high anatomical accuracy of the model.

ii. Biomechanical validation:

Finite element analysis was performed on the aforementioned pelvic model. The bilateral acetabula were fixed to restrict their free movement in six degrees of freedom. A load of 600 N was applied along the gravitational direction on the upper surface of the first sacral vertebra (S1) to simulate the standing posture. Through the analysis, the displacement and stress distribution characteristics of the model were obtained. These characteristics were compared with those reported in previous literature to verify the effectiveness of the finite element model (Hu et al., 2019).

### 2.4 Sacral fracture and internal fixation device modeling

Based on the validated pelvic finite element model, an unstable Denis zone I sacral fracture was created using the “Split” function in SolidWorks 2022 (Dassault Systèmes, France). A vertical fracture line was generated through the sacral ala in accordance with the Denis classification (Gierig et al., 2021) (Figure 2b). All three-dimensional models of the internal fixation devices were designated in SolidWorks 2022 using parametric modeling techniques. The geometric dimensions were defined strictly according to the manufacturer’s specifications and verified through physical measurements to ensure dimensional precision (Supplementary Table S1) (Figures 2c,e). The final assembled models of the pedicle screw–K-wire construct and the SIJS system are presented (Figures 2d,f).

### 2.5 Assembly of the sacral fracture internal fixation model

For finite element analysis, the established Denis zone I sacral fracture model was virtually assembled with two internal fixation systems using SolidWorks 2022 (Dassault Systèmes, France). For Group A (P-INFIX), the pedicle screws, transverse K-wire, and fractured pelvic model were imported into the assembly. To simulate postoperative biomechanics, the contact between fracture surfaces was defined as “frictionless,” representing the potential for micro-motion immediately after surgery. All bone–implant interfaces were defined as “bonded” to stimulate secure osseous fixation during the early postoperative period. The completed assembly was exported in X-T format. For Group B (SIJS), identical assembly procedures and contact settings were applied. The SIJS construct was assembled with the fractured pelvic model, with the fracture surface defined as “frictionless” and the bone–screw interface defined as tied. The final assembly was similarly exported in X-T format for subsequent analysis.

### 2.6 Finite element analysis of two different fixations for denis I sacral fracture

The X-T format assemblies for Groups A and B were imported into ANSYS 2024R1 (ANSYS, Inc., United States) (Kozaki et al., 2023). The analysis sequence included material assignment, contact definition, ligament spring addition, meshing, load application, and boundary condition setup. Pelvic finite element models were analyzed under sitting, supine, and lateral decubitus conditions. The loading and constraint conditions were defined as follows:

- Sitting position: A “coupling” constraint was applied to the superior surface of the first sacral vertebra. A 400 N vertical load was applied downward to simulate upper body weight, and all six degrees of freedom were restricted for both acetabula.
- Lateral decubitus position: A “coupling” constraint was applied to the iliac crest on the affected side. A 400 N horizontal load was applied towards the unaffected side, with all six degrees of freedom restricted for the unaffected acetabulum.
- Supine position: A “coupling” constraint was applied to the pubic surface. A 400 N load was applied posteriorly toward the sacrum, with all six degrees of freedom restricted at the superior and inferior sacral ends.

Observation parameters included:

1. Fracture surface displacement: Nodal displacement data were collected along the medial and lateral edges of the sacral fracture surface and labeled as Z1, Z2 (sitting), Y1, Y2 (supine), and C1, C2 (lateral decubitus). These values were statistically analyzed, with greater displacement indicating reduced fixation stability.
2. Implant stress distribution: stress contour plot were used to identify areas of stress concentration and maximum stress values within the fixation devices. A higher stress concentration indicated a greater likelihood of implant loosening or fatigue fracture.

### 2.7 Statistical analysis

All statistical analyses were conducted using SPSS version 26.0 (IBM Corp, United States). Normally distributed continuous data were expressed as mean ± standard deviation and compared between groups using the independent-sample t-test. Non-normally distributed data were presented as median (interquartile range) and compared using the Mann-Whitney U test. A P value <0.05 was considered statistical significant.

## 3 Results

### 3.1 Validation of the pelvic finite element model

**FIGURE 3:**
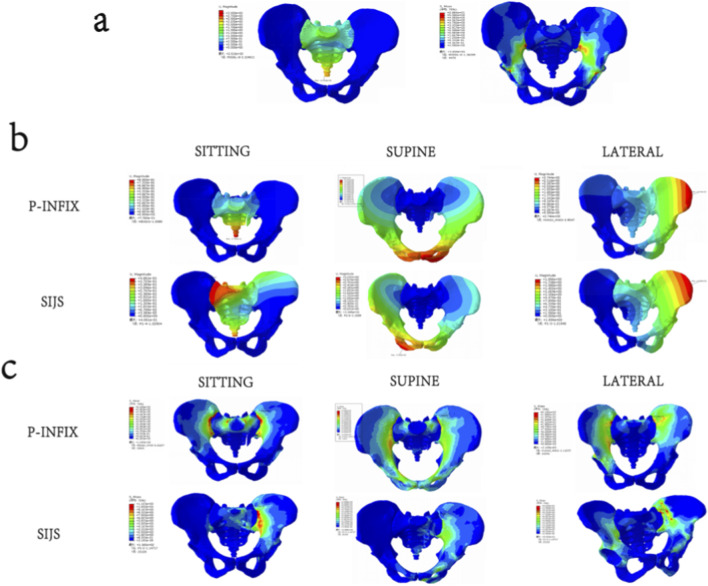
Stress and strain results of the pelvis implanted with P-INFIX and SIJS devices under different loading conditions. **(a)** Stress and strain contour plots of the intact pelvis under physiological loading. **(b)** Stress contour plots of the pelvis with P-INFIX and SIJS devices in sitting, supine, and lateral positions. **(c)** Strain contour plots of the pelvis with P-INFIX and SIJS devices in sitting, supine, and lateral positions.

After constructing the three-dimensional pelvic geometric model, we compared the paired measurements of key anatomical distances between the finite element model and the volunteer’s CT data. The discrepancies at all measurement points fell within an acceptable range, indicating that the model accurately reproduced the real anatomical structure (Supplementary Table S2). When a static load was applied to the standard pelvic model, its displacement and stress distributions exhibited characteristics consistent with physiological symmetry (Figure 3a). The peak displacement occurred at the anterior edge of the coccyx, while stress was transmitted along the sacrum-sacroiliac joint-iliac bone pathway, with a maximum stress value of 38.84 MPa. These distribution patterns are highly consistent with established biomechanical principles and previous research findings (Zhao et al., 2025), confirming that this model is suitable for subsequent fracture analysis and internal fixation studies.

### 3.2 Biomechanical performance evaluation of the two internal fixation systems under different positions

**FIGURE 4:**
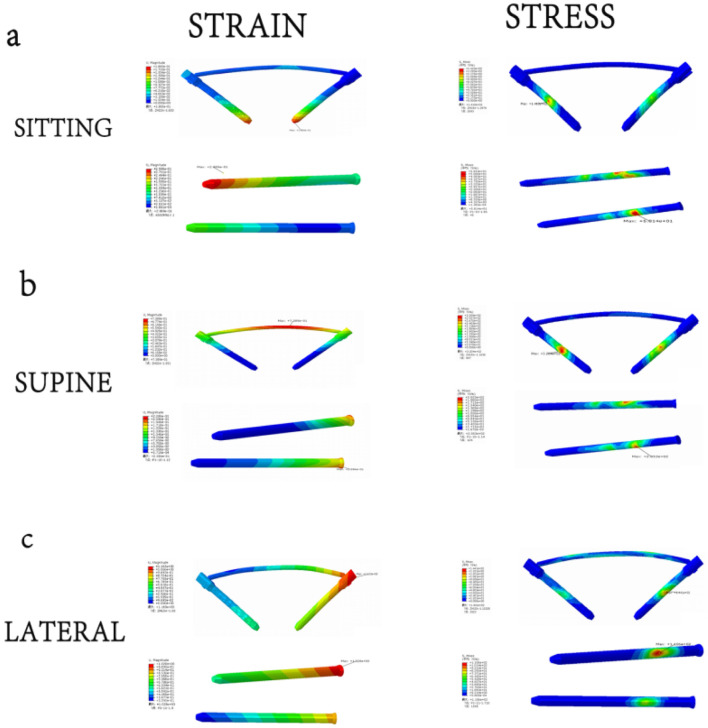
Stress and strain nephograms of P-INFIX and SIJS devices under different working conditions. **(a)** Stress and strain nephograms of P-INFIX and SIJS devices in sitting position. **(b)** Stress and strain nephograms of P-INFIX and SIJS devices in supine position. **(c)** Stress and strain nephograms of P-INFIX and SIJS devices in lateral position.

This study systematically evaluated the biomechanical performance of the novel P-INFIX system (Group A) and the SIJS (Group B) under three physiological positions. Overall, the P-INFIX system demonstrated biomechanical performance comparable to that of the SIJS under most test conditions, while exhibiting certain advantages in structural stability and fracture fragment control in specific directions. (Figures 3b,c). The stress levels of all fixation devices remained within the safe range of the material (Figure 4). The summarized results are presented with detailed analyses as follows (Tables 3, 4).

**TABLE 3:** Fracture fragment displacement distance.

Study Group	SITTING				SUPINE				LATERAL			
	N	Z1	N	Z2	N	Y1	N	Y2	N	C1	N	C2
A组	118	0.12 (0.08,0.23)	135	0.18 (0.11,0.21)	58	1.06 (0.81,1.28)	88	0.01 (0.01,0.02)	89	0.68 (0.65,0.74)	112	0.66 (0.65,0.68)
B组	39	0.11 (0.06,0.16)	41	0.13 (0.09,0.18)	39	0.13 (0.05,0.17)	41	0.03 (0.02,0.04)	39	0.578 (0.54,0.68)	41	0.59 (0.53,0.76)
t/z值	−2.242		−3.295		−8.321		−5.078		−4.527		−2.484	
P	0.025*		0.001*		<0.001*		<0.001*		<0.001*		0.013*	

The bold values indicates statistically significant difference, *P<0.05.

**TABLE 4:** Maximum displacement and stress values of internal fixation.

Position	Maximum displacement (mm)		Maximum stress (MPa)	
	Group A (P-INFIX)	Group B (SIJS)	Group A (P-INFIX)	Group B (SIJS)
Sitting position	0.18	0.29	141	58
Supine position	0.78	0.22	320	205
Lateral position	1.16	1.02	144	110

#### 3.2.1 Biomechanical performance in the sitting position

In the sitting position, SIJS (Group B) provided better control of lateral fracture fragment displacement (Z2) compared to P-INFIX (Group A) (P = 0.001). However, the difference in medial fracture fragment stability (Z1) between the two groups was not significant (P = 0.025). All displacement values were low (Group A: 0.12–0.18 mm; Group B: 0.11–0.13 mm), indicating that both systems provided satisfactory clinical fixation. Regarding device performance, the maximum displacement of Group A (P-INFIX) was smaller than that of Group B (0.18 mm vs. 0.29 mm). Stress analysis showed that the peak stress of Group A was 141 MPa, higher than that of Group B (58 MPa); however, both were far below the yield limit of the titanium alloy, indicating a sufficient safety margin.

#### 3.2.2 Biomechanical performance in the supine position

In the supine position, SIJS (Group B) was superior to P-INFIX (Group A) in controlling lateral fracture fragment displacement (Y1), with a displacement difference of 0.93 mm (P < 0.001). In contrast, Group A performed better in controlling medial fracture fragment displacement (Y2), with a displacement difference of 0.02 mm (P < 0.001). Regarding device performance, overall displacement in Group B was lower than in Group A (0.22 mm vs. 0.73 mm). The peak stress of Group A reached 320 MPa, higher than that of Group B (205 MPa); however, both values remained within the material’s safe range.

#### 3.2.3 Biomechanical performance in the lateral decubitus position

In the lateral decubitus position, SIJS (Group B) was superior to P-INFIX (Group A) in controlling both medial (C1) and lateral (C2) fracture fragment displacements, with displacement differences of 0.11 mm (P < 0.001) and 0.07 mm (P = 0.013), respectively. Regarding device performance, the maximum displacement values of both groups were similar (Group A: 1.16 mm; Group B: 1.02 mm). The peak stress was 144 MPa for Group A and 110 MPa for Group B. In both groups, stress concentration was primarily located in the middle-posterior segment of the screws, and stress levels remained within the material’s safe range.

## 4 Discussion

This finite element analysis demonstrates that the novel internal fixation system (P-INFIX) provides biomechanical performance comparable to that of the gold standard SIJS for treating Denis zone I sacral fractures, and theoretically reduces the risk of neurovascular injury through its innovative and safer surgical pathway. The combination of mechanical effectiveness and procedural safety makes P-INFIX a promising alternative to SIJS, particularly in resource-limited primary care settings. Both the SacroNail system (Marintschev and Hofmann, 2023) and the SacralBar construct (Wagner et al., 2021) are capable of providing satisfactory stability for posterior pelvic ring fixation. Nevertheless, implantation of the SacroNail requires positioning and fixation assistance from specialized instruments, which limits its popularization in primary hospitals. Moreover, to enhance the stability of the sacroiliac joint, supplementary SIJS fixation is still necessary in certain cases treated with the SacralBar technique.

From a technical standpoint, the finite element model developed through multisoftware integration in this study successfully simulated biomechanical responses under physiological loading. The results showed that P-INFIX maintained overall pelvic stability comparable to SIJS. This can be attributed to its frame-like design, in which bilateral screws and a transverse connecting structure establish an effective load-sharing mechanism that supports the pelvic ring similarly to SIJS (Huang et al., 2024). The posterior pelvic ring accounts for approximately 70% of the overall pelvic stability, serving as the core anatomical structure to resist vertical loads, shear forces and rotational stresses (Zhou and Cheng, 2025). The anterior pelvic ring primarily acts as a tension band for auxiliary stabilization, mainly counteracting pelvic external rotation and diastatic stresses (Gänsslen et al., 2025). Notably, the frame structure of the P-INFIX system not only stabilizes the posterior pelvic ring, but also distributes loads bilaterally to the iliac bones via transverse connection, which indirectly reduces the tensile load borne by the anterior pelvic ring (including the pubic rami and symphysis pubis). Unlike conventional sacroiliac joint screws (SIJS), which only achieve compression of the posterior pelvic ring, the P-INFIX system exhibits potential advantages in patients combined with anterior pelvic ring injuries.

A load of 400 N accounts for 50%–60% of body weight, which is physiologically relevant for individuals weighing 70–80 kg (Wang et al., 2015; Carp et al., 2026). Regarding fracture fragment stability, although SIJS demonstrated a statistical advantage in controlling lateral fragment displacement, the clinical relevance of this difference is minimal. All displacement values were far below the critical clinical threshold of 10 mm (Noureldine et al., 2023), indicating that both fixation systems provided sufficient mechanical stability for fracture healing. Notably, P-INFIX performed comparably to SIJS in controlling medial fragment displacement, underscoring the advantage of its frame structure in maintaining overall alignment. In terms of implant performance, although the peak stress in P-INFIX was higher than that in SIJS, it remained well below the yield strength of titanium alloy (1,050 MPa) (Li et al., 2024), confirming an adequate safety margin under physiological loading. The stress concentration areas in P-INFIX provide valuable insight for future structural optimization; in contrast, the uniform stress distribution in SIJS reflects the maturity and stability of this established technique. Biomechanically, the performance of P-INFIX under different loading positions aligns with its design rationale: its superior performance in the sitting position reflects efficient load sharing through the frame structure; in contrast, the stress patterns in the lateral decubitus position reveal its response to torsional stress. These findings confirm the feasibility of P-INFIX and provide a foundation for further refinement.

Mechanical analysis of the implants revealed that although the peak stress of the P-INFIX construct was higher than that of sacroiliac screws, it remained far below the yield strength of titanium alloy (1,050 MPa), indicating an adequate safety margin for the implant system. Obvious discrepancies in implant stress were observed across different body positions: the peak stress of P-INFIX reached 320 MPa in the supine position, nearly twice the value measured in the sitting position (141 MPa). This discrepancy can be fundamentally explained by the load characteristics under supine posture: loads transmit posteriorly in the supine position, exerting continuous compression on the posterior sacrum and transverse Kirschner wires, which leads to localized stress concentration and bending deformation of the wires. Based on the above biomechanical findings, we recommend that patients adopt a semi-recumbent or lateral position in the early postoperative period to reduce excessive stress borne by the implants and preserve fixation stability.

More importantly, the core value of the present study lies in addressing the long-standing drawbacks of conventional posterior pelvic ring fixation techniques. Sacroiliac joint screw (SIJS) placement heavily relies on the surgeon’s experience and high-end navigation equipment, leading to substantial radiation exposure for both patients and operators, as well as an elevated risk of neurovascular injury (Li N. et al., 2023). In contrast, the P-INFIX system adopts a safe screw insertion trajectory lateral to the posterior superior iliac spine (PSIS). This intuitive surgical approach reduces reliance on continuous fluoroscopic guidance, shortens the learning curve, and enables safe implementation without sophisticated equipment, making it particularly suitable for primary hospitals. Furthermore, compared with compression locking plates, the subcutaneous transverse connector of the P-INFIX system minimizes postoperative discomfort and the risk of pressure ulcers induced by prominent implants (Zhang et al., 2022). Unlike lumbopelvic fixation, the P-INFIX construct does not traverse the lumbosacral junction, which preserves lumbar spinal mobility and lowers the long-term risk of implant fatigue failure (Chaiyamongkol et al., 2018). Additionally, it avoids soft tissue irritation and lumbosacral plexus injuries frequently associated with traditional techniques such as sacral bars (Wagner et al., 2021). Collectively, these design innovations represent a paradigm shift: without compromising biomechanical efficacy, design-INFIX transforms posterior pelvic ring fixation from a highly specialized and equipment-dependent procedure into a safer, standardized, and more accessible technique. The biomechanical evidence presented in this study provides a strong foundation for its clinical translation, especially in resource-constrained healthcare settings.

This study has several limitations. First, the finite element analysis utilized linear elastic material properties, which cannot fully replicate the nonlinear mechanical characteristics of bones and ligaments. Besides, the mechanical effects of muscles and soft tissues were excluded, leading to deviations from the actual *in vivo* biomechanical environment. Second, the anatomical model was reconstructed from CT data of a single healthy male volunteer, which limits its representativeness for females, patients with osteoporosis, and individuals with sacral malformations. Third, only immediate postoperative static loading was analyzed, without considering long-term cyclic loading and implant fatigue, failing to fully evaluate the long-term stability of the fixation system. Furthermore, the sizes of screws and transverse rods were selected based on the conventional anterior INFIX system, and no comparison of implants with different dimensions was conducted. In addition, the single-model design constrains the generalizability of the findings, and further validation with multiple diversified models is required.

## 5 Conclusion

In conclusion, this finite element analysis demonstrated that the novel P-INFIX system offers biomechanical performance comparable to SIJS for Denis zone I sacral fractures, satisfying clinical requirements for both fracture stability and implant safety. Owing to its innovative and safe surgical approach, P-INFIX reduces the learning curve, minimizes intraoperative radiation exposure, and lowers the risk of neurovascular injury. These advantages establish P-INFIX as a safe, effective, and practical option for managing unstable posterior pelvic ring injuries, providing critical evidence to support its broader clinical adoption. Future studies should focus on fatigue testing, cadaveric validation, and clinical trials to comprehensively assess its long-term performance and facilitate its integration into routine clinical practice.

## Data Availability

The original contributions presented in the study are included in the article/Supplementary Material, further inquiries can be directed to the corresponding authors.
