# Supplementary material for: A novel internal fixation system for sacral fractures: a finite element biomechanical study
Source: Front Bioeng Biotechnol. 2026 Jul 10;14:1871603. doi: 10.3389/fbioe.2026.1871603 (PMC13395768; doi:10.3389/fbioe.2026.1871603)
Supplement: Supplementary file 1 [file DataSheet1.docx]

| **Material** | Length（mm） | Diameter（mm） |
| --- | --- | --- |
| Pedicle Screw | 50 | 6.0 |
| K-wire | 63 | 4.0 |
| Iliac Screw | 75 | 7.0 |

**Supplement Table1. Dimension parameter of FEA models**

| **Model** | a | b | c | d | e | f | g | h | i | j |
| --- | --- | --- | --- | --- | --- | --- | --- | --- | --- | --- |
| **Finite Element Model** | 107.36 | 34.46 | 118.45 | 106.36 | 120.88 | 50.87 | 59.26 | 116.29 | 142.38 | 37.75 |
| **Actual CT Data** | 107.25 | 34.45 | 118.40 | 105.95 | 120.78 | 50.85 | 59.21 | 116.25 | 142.10 | 37.70 |

**Supplementary Table 2. Distance Parameters (mm) of Marked Points Between Pelvic Finite Element Model and Actual Pelvic CT Data**


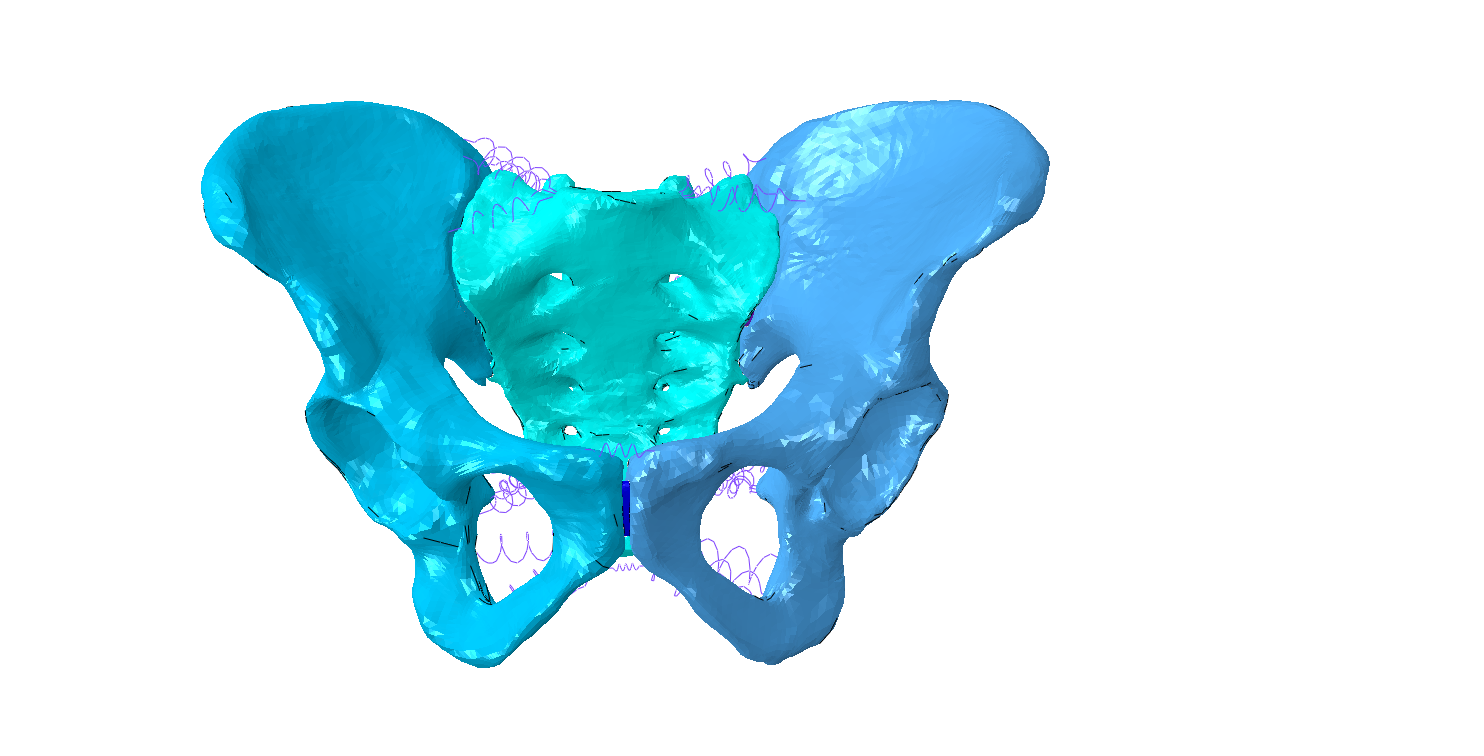


**Supplement Figure 1 Ligament Structure Diagram of the Finite Element Model**
